# High-efficiency production of the antimicrobial peptide pediocin PA-1 in metabolically engineered *Corynebacterium glutamicum* using a microaerobic process at acidic pH and elevated levels of bivalent calcium ions

**DOI:** 10.1186/s12934-023-02044-y

**Published:** 2023-02-27

**Authors:** Jens Christmann, Peng Cao, Judith Becker, Christian K. Desiderato, Oliver Goldbeck, Christian U. Riedel, Michael Kohlstedt, Christoph Wittmann

**Affiliations:** 1 Institute for Systems Biotechnology, Saarland University, Saarbrücken, Germany; 2 Institute of Microbiology and Biotechnology, University of Ulm, Ulm, Germany

**Keywords:** *Corynebacterium glutamicum*, Pediocin PA-1, Bioprocess, Food additive, Bacteriocin, Antimicrobial peptide, Acidic pH, Oxygen limitation, Calcium supplementation, *Listeria sp*

## Abstract

**Background:**

Pediocin PA-1 is a bacteriocin of recognized value with applications in food bio-preservation and the medical sector for the prevention of infection. To date, industrial manufacturing of pediocin PA-1 is limited by high cost and low-performance. The recent establishment of the biotechnological workhorse *Corynebacterium glutamicum* as recombinant host for pediocin PA-1 synthesis displays a promising starting point towards more efficient production.

**Results:**

Here, we optimized the fermentative production process. Following successful simplification of the production medium, we carefully investigated the impact of dissolved oxygen, pH value, and the presence of bivalent calcium ions on pediocin production. It turned out that the formation of the peptide was strongly supported by an acidic pH of 5.7 and microaerobic conditions at a dissolved oxygen level of 2.5%. Furthermore, elevated levels of CaCl_2_ boosted production. The IPTG-inducible producer *C. glutamicum* CR099 *pXMJ19* *P*_*tac*_* pedACD*^*Cg*^ provided 66 mg L^−1^ of pediocin PA-1 in a two-phase batch process using the optimized set-up. In addition, the novel constitutive strain *P*_*tuf*_* pedACD*^*Cg*^ allowed successful production without the need for IPTG.

**Conclusions:**

The achieved pediocin titer surpasses previous efforts in various microbes up to almost seven-fold, providing a valuable step to further explore and develop this important bacteriocin. In addition to its high biosynthetic performance *C. glutamicum* proved to be highly robust under the demanding producing conditions, suggesting its further use as host for bacteriocin production.

**Supplementary Information:**

The online version contains supplementary material available at 10.1186/s12934-023-02044-y.

## Background

Antimicrobial peptides (AMPs) are a group of bioactive substances that induce transmembrane pores and attack other intracellular targets, capable of rapidly killing microorganisms [1]. The typically small molecules are ribosomally synthesized by various cell types and tissues, including invertebrates, plants, animals, and last not least, a wide range of bacteria, AMPs of the latter being classified as bacteriocins [2, 3]. In contrast to most currently available antibiotics, many bacteriocins have only narrow spectra of target organisms and are therefore regarded attractive agents for precision therapy and prevention of infection [4]. Furthermore, bacteriocins have become important and safe agents for food preservation, because they efficiently act against food borne pathogens, while their peptide structures are easily digested in the human body [5]. Bacteriocins have recognized commercial relevance, as foodborne illness causes to 420,000 annual deaths worldwide [6] and two-third of food related diseases are caused by bacterial contamination [7].

Pediocins are commercially relevant bacteriocins of the class IIa type [8, 9]. They have been shown to efficiently bind to their receptors in bacterial membranes resulting in pore formation and killing of the target cell by destruction of the proton motive force [10, 11]. They efficiently act against *Listeria monocytogenes*, a food-borne pathogen of increasing concern to the food industry which may be found in raw milk, dairy products, vegetables, and meat products and can grow under refrigeration temperatures (growth has been reported at temperatures as low as − 1 °C), high salt concentrations (up to 10%), low pH (pH 5.0), and high temperatures (44 °C) [12]. Native pediocin producers are lactic acid bacteria including species of *Pediococcus* [13] and *Lactobacillus* [14]*.* The most prominent pediocin is pediocin PA-1, produced by *Pediococcus acidilactici* and extensively studied over the past decades due to its unique properties and application potential [15], including identification and sequencing of the encoding pediocin PA-1 cluster *pedABCD* [13, 14, 16, 17].

A commercial pediocin formulation (non-purified fermentates of native food grade producers with low levels of the antimicrobial peptide) has been introduced under the trade name ALTA (Quest International, Irvine, CA, USA) [18]. However, pure pediocin PA-1 is not commercially available [19]. For research purposes, concentrated bacteriocin solutions are generally produced by costly cultivation of natural producers and subsequent peptide purification [20, 21]. The efficiency of production is limited by the need for expensive media (that meet the multiple auxotrophies of the native producer strains) and the ultimately low titer [22]. To enable a broader access to pediocins for food applications but also clinical trials, production processes with higher pediocin titers and reduced costs appear crucial [23–25]. For further development, the successful demonstration of heterologous pediocin PA-1 production in the Gram-positive soil bacterium *Corynebacterium* *glutamicum* (upon episomal expression of a codon optimized and shortened version of the pediocin PA-1 cluster (*pedACD*) under induction by IPTG) recently provided a valuable and promising starting point [23].

Here, we advanced the production of pediocin PA-1 using the recently developed producer *C. glutamicum* CR099 *pXMJ19 P*_*tac*_* pedACD*^*Cg*^. The microbe turned out to be highly robust to the production and the presence of the peptide. Several rounds of medium and bioprocess optimization finally provided an optimum operation mode at slightly acidic pH, limiting oxygen supply, and elevated levels of calcium ions in a lean production medium. Benchmarked in a batch process, the microbe produced pediocin PA-1 up to 135,700 BU mL^−1^ (66 mg L^−1^), almost seven-fold more than any other producers studied so far. A novel *C. glutamicum* strain that expressed the cluster under control of the constitutive *P*_*tuf*_ promotor enabled pediocin PA-1 production up to 82,800 BU mL^−1^ without the need for costly IPTG. Our work displays a valuable demonstration of using *C. glutamicum* in an advanced process setting for high-level pediocin PA-1 production, providing this important bioactive for further exploration and promising to use the host also for other bacteriocins in the future.

## Results

### *C. glutamicum* appears metabolically unaffected by production of the antimicrobial peptide pediocin PA-1

**Fig. 1:**
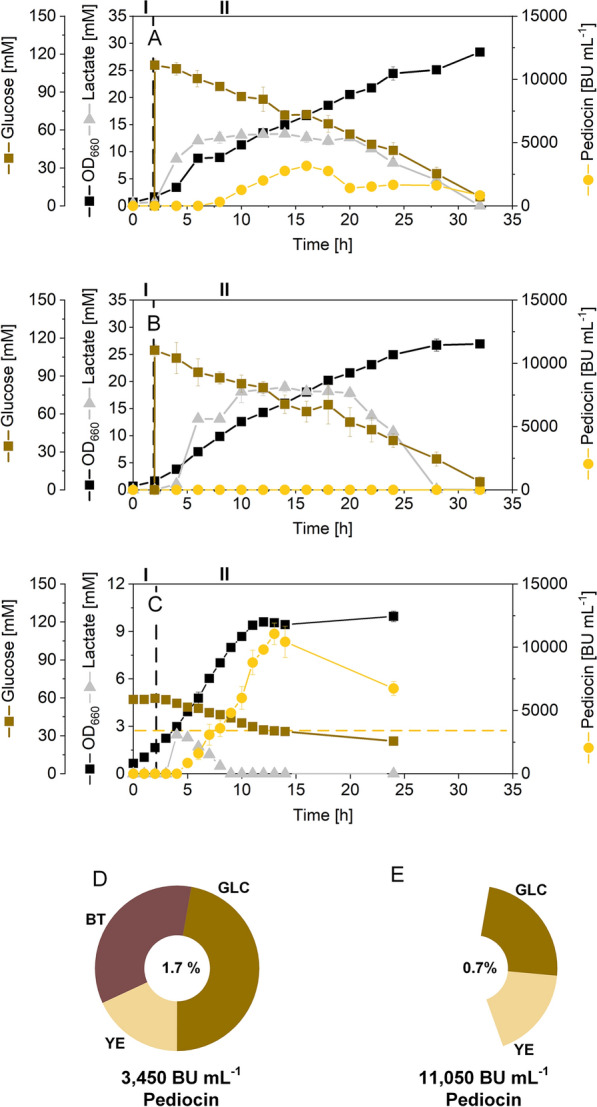
Pediocin production in shake flasks using recombinant *C. glutamicum* CR099* pXMJ19 P*_*tac*_* pedACD*^*Cg*^. The data show growth and production on TY medium, containing 20 g L^−1^ of yeast extract, 16 g L^−1^ of bacto-tryptone, additionally supplemented with 20 g L^−1^ of glucose and 0.2 mM of IPTG after 2 h, indicated by a vertical dashed line **A**. The non-producing reference strain *C. glutamicum* CR099 *pXMJ19*, expressing the empty vector, is shown for comparison (**B**). In addition, GY medium was used to produce pediocin (**C**). The medium contained 10 g L^−1^ of glucose, 10 g L^−1^ of yeast extract, mineral salts, and vitamins. It was supplemented with IPTG (0.2 mM), two hours after the start. In addition, the corresponding total carbon content of the TY (**D**) and the GY medium (**E**) is given. For the complex ingredients, the total carbon content was estimated from the composition given by the supplier, whereby carbohydrates were considered as glucose units [34]. n = 3

Recently, we demonstrated production of the antimicrobial peptide pediocin PA-1 using *C. glutamicum* CR099 *pXMJ19 P*_*tac*_* pedACD*^*Cg*^ that expressed a codon-optimized version of the pediocin PA-1 cluster from *P. acidilactici* under control of the inducible *P*_*tac*_ promotor [23]. The cluster encoded the pre-pediocin PA‐1 (PedA), the accessory export protein PedC, and the ABC transporter PedD (involved in the excision of the signal peptide and the secretion of pediocin) respectively [26]. Incubated on TY medium, the mutant started to grow from early on (Fig. 1A). After induction with IPTG, it took about 5 h until pediocin PA-1 became detectable in the culture broth. The level of the peptide reached a maximum of 3450 BU mL^−1^ after 16 h (i. e. 11 h after induction). During later stages, the concentration of the peptide dropped to finally below 828 BU mL^−1^. Throughout the process, the cells grew linearly up to a final OD_660_ of 28.4. Meanwhile, glucose was continuously consumed. After 32 h, the sugar was fully depleted. The control strain expressing the empty plasmid, did not produce any pediocin PA-1, as expected (Fig. 1B). In terms of growth and substrate consumption, it behaved exactly as strain *C. glutamicum* CR099 *pXMJ19 P*_*tac*_* pedACD*^*Cg*^. On a first glance, this suggested that the microbe seemed not affected by the recombinant pathway and, also, not by the antimicrobial product itself.

We now were interested to assess the influence of pediocin PA-1 on *C. glutamicum* in more detail and compared the transcriptome of the producer to that of the control strain. The two strains were sampled at 13 h of the process, reflecting a time point, where the producing cells actively synthetized pediocin PA-1 and already faced a higher level of the product. The transcriptome data revealed excellent quality and reproducibility among the biological replicates (Additional file 1: Fig. S1). The pediocin cluster genes were strongly up regulated in the producer, indicating efficient transcription of the heterologous pathway (Table 1). Regarding the native metabolism of *C. glutamicum*, the impact of pediocin PA-1 production on gene expression was rather small. Only very few out of more than 3000 genes were significantly changed in expression in the producer, as compared to the reference strain (Table 1). The gene *CGL_RS05840,* encoding a pspC binding domain containing protein from the group of phage shock proteins as response to cell envelope stress [27, 28] was up regulated. In contrast, *bioY*, encoding a biotin transporter*,* was found down regulated. A few other genes were weakly affected in expression. Taken together, *C. glutamicum* CR099 *pXMJ19 P*_*tac*_* pedACD*^*Cg*^ tolerated the implemented pediocin PA-1 production well. The microbe appeared vital in terms of growth and, also, the global transcriptional machinery remained almost unchanged. Severe bottlenecks in metabolism did not show up, suggesting focusing on the bioprocess level for further improvement rather than on engineering the cell factory.

**Table 1:** Strains and plasmids used in this study

Strain	Description	Refs.
*E. coli*		
DH10B	Cloning host	[98]
*L. innocua*		
*pIMK2*	Sensor strain expressing the plasmid *pIMK2 kan*^*R*^	[23]
*pNZ44*	Sensor strain expressing the plasmid *pNZ44 cm*^*R*^	[23]
*C. glutamicum*		
CR099	Genome reduced derivative of strain ATCC 13,032 with deletion of *ΔCGP1-3* and* ΔISCg1-2*	[96]
CR099 *pEKEx2 P*_*tac*_* pedACD*^*Cg*^	CR099 with episomal expression of codon-optimized pediocin operon *pedACD*^*Cg*^ from *Pediococcus acidilactici* under control of *P*_*tac*_	[23]
CR099 *pXMJ19 P*_*tac*_* pedACD*^*Cg*^	CR099 with episomal expression of the codon-optimized pediocin operon from *P. acidilactici* under control of *P*_*tac*_	[23]
CR099* pXMJ19*	CR099 with episomal expression of empty *pXMJ19*	[23]
CR099 *pClik 5α P*_*tuf*_* pedACD*^*Cg*^	CR099 with episomal expression of the codon-optimized pediocin operon from *P. acidilactici* under control of *P*_*tuf*_	This work
CR099 *pEKEx2 P*_*tac*_* pedA*^*M31L*^*CD*^*Cg*^	CR099 with episomal expression of the codon-optimized pediocin operon from *P. acidilactici* under control of *P*_*tac*_ including mutated *pedA*^*M31L*^	This work
CR099* pClik 5α*	CR099 with episomal expression of empty *pClik 5α*	This work
CR099* pClik 5α vgb*	CR099 with episomal expression of the heterologous gene *vgb* from *Vitreoscilla* spp. [52] under control of *P*_*tuf*_	This work
CR099* pClik 5α dps*	CR099 with episomal expression of the native *C. glutamicum* gene *dps* [49] under control of *P*_*tuf*_	This work
CR099* pClik 5α mcbR*	CR099 with episomal expression of the native *C. glutamicum* gene *mcbR* [49] under control *P*_*tuf*_	This work
CR099* pClik 5α mpx*	CR099 with episomal expression of the native *C. glutamicum* gene *mpx* [47] under control of *P*_*tuf*_	This work
CR099* pClik 5α mshA*	CR099 with episomal expression of the native *C. glutamicum* gene *mshA* [48] under control of *P*_*tuf*_	This work
CR099* pClik 5α katA*	CR099 with episomal expression of the native *C. glutamicum* gene *katA* [49] under control of *P*_*tuf*_	This work
CR099* pClik 5α zwf*	CR099 with episomal expression of the native *C. glutamicum* gene *zwf* [99] under control of *P*_*tuf*_	This work
CR099 *pClik 5α zwf *^*ATG*^	CR099 with episomal expression of the native *C. glutamicum* gene *zwf* under control of the translational start codon ATG [99] and *P*_*tuf*_	This work
CR099 *pClik 5α ATG zwf *^*ATG, A243T*^	CR099 with episomal expression of the mutated variant z*wf *^*A243T*^ [56] under control of the translational start codon ATG [99] and *P*_*tuf*_	This work
CR099 *pClik 5α P*_*H30*_ *DR1558*	CR099 with episomal expression of the heterologous gene *DR1558 from Deinococcus radiodurans* [50] under control of the synthetic promotor *P*_*H30*_	This work
CR099 *pClik 5α P*_*tuf*_* DR1558*	CR099 with episomal expression of the heterologous gene *DR1558 from Deinococcus radiodurans* [50] under control of *P*_*tuf*_	This work
Plasmids		
*pClik5α* MCS	Episomal vector, ORI^*Cg*^, ORI^*Ec*^, k*an*^*R*^	[97]
*pXMJ19*	Episomal vector, *P*_*tacI*_* lacI*, ORI^*Cg*^, ORI^*Ec*^, *cm*^*R*^	[23]
*pXMJ19 P*_*tac*_* pedACD*^*Cg*^	Episomal expression of *pedACD*^*Cg*^ under control of *P*_*tac*_	[23]
*pClik 5α P*_*tuf*_* pedACD*^*Cg*^	Episomal expression of *pedACD*^*Cg*^ under control of *P*_*tuf*_	This work
*pClik 5α P*_*tuf*_* vgb*	Episomal expression of *vgb* [52] under control of *P*_*tuf*_	This work
*pClik 5α P*_*tuf*_* dps*	Episomal expression of *dps* [49] under control of *P*_*tuf*_	This work
*pClik 5α P*_*tuf*_* mcbR*	Episomal expression of *mcbR* [49] under control of *P*_*tuf*_	This work
*pClik 5α P*_*tuf*_* mpx*	Episomal expression of *mpx* [47] under control of *P*_*tuf*_	This work
*pClik 5α P*_*tuf*_* mshA*	Episomal expression of *mshA* [48] under control of *P*_*tuf*_	This work
*pClik 5α P*_*tuf*_* katA*	Episomal expression of *katA* [49] under control of *P*_*tuf*_	This work
*pClik 5α P*_*tuf*_* zwf*	Episomal expression of *zwf* under control of *P*_*tuf*_	This work
*pClik 5α P*_*tuf*_* zwf *^*ATG*^	Episomal expression of *zwf* under control of the start codon ATG [12] and *P*_*tuf*_	This work
*pClik 5α P*_*tuf*_* zwf *^*ATG, A243T*^	Episomal expression of the mutated variant *zwf *^*A243T*^ [56] under control of the start codon ATG [12] and *P*_*tuf*_	This work
*pClik 5α P*_*H30*_* DR1558*	Episomal expression of *DR1558* [50] under control of *P*_*H30*_	This work
*pClik 5α P*_*tuf*_* DR1558*	Episomal expression of *DR1558* [50] under control of *P*_*tuf*_	This work

### Fine-tuning of oxygen supply is crucial to preserve active pediocin PA-1 while enabling growth of the aerobic microbe

Pediocin PA-1 contained an oxygen-sensitive l-methionine residue at position 31, and oxidation of this amino acid residue was known to cause a loss of antimicrobial activity [19]. To overcome this problem, we first followed a previous strategy that had demonstrated increased stability of mutated peptide variants in which the l-methionine residue had been replaced by small nonpolar amino acids, including l-leucine [29]. In short, we created the pediocin PA-1 variant *pedA*^*M31L*^ using a mutated primer that carried the desired point mutation. After validation by sequencing, the novel strain *pEKEx2 P*_*tac*_* pedA*^*M31L*^*CD*^*Cg*^, based on a well-established shuttle vector for expression in *C. glutamicum* [30], was cultivated as described above. Unfortunately, the alteration of the peptide did not provide any detectable active pediocin PA-1 (Additional file 1: Fig. S3).

**Fig. 2:**
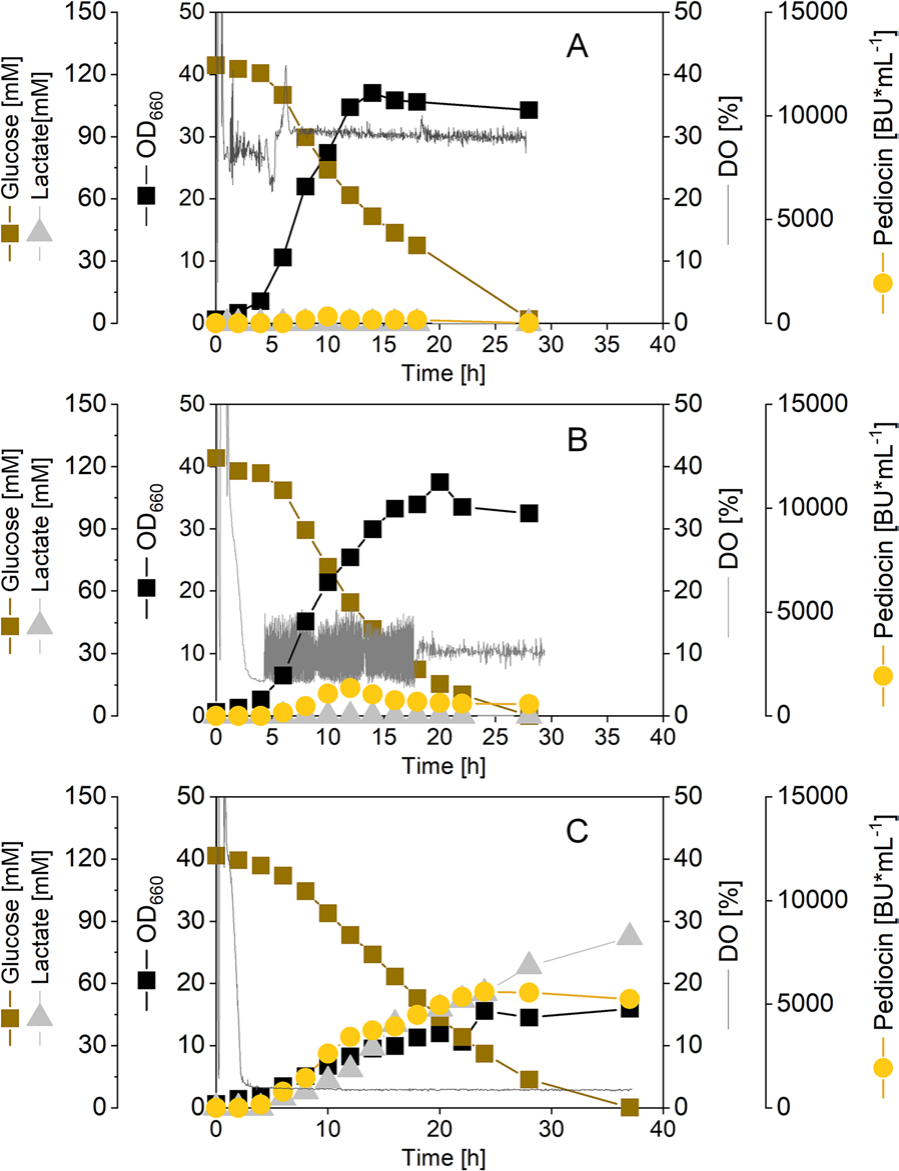
Impact of the dissolved oxygen (DO) level on pediocin production in recombinant *C. glutamicum* CR099* pXMJ19 P*_*tac*_* pedACD*^*Cg*^. The process was conducted in lab scale bioreactors using rich TY medium, supplemented after two h with 20 g L^−1^ of glucose (pH 6.5 and 30 °C). The DO level was controlled at 30% (A), 10% (**B**), and 2.5% (**C**). The experiments were performed as single replicate each. The conducted processes, according to experience, have a standard deviation of less than 10%

At this point, we decided to keep producing the native pediocin PA-1 molecule and optimize the oxygen supply instead. For this purpose, the production process was transferred to lab scale bioreactors, which allowed a precise adjustment of the dissolved oxygen (DO) level by the stirring and the aeration rate. In addition to a well-supplied reactor (DO level of 30%), two set-ups were conducted at lower DO levels (5% and 2.5%) (Fig. 2A–C). As expected, the oxygen availability strongly affected the growth of *C. glutamicum* CR099 *pXMJ19 P*_*tac*_* pedACD*^*Cg*^. The cells grew best at the highest oxygen supply (µ = 0.45 h^−1^), still managed well at 5% (µ = 0.41 h^−1^) but were found reduced in growth at 2.5% (µ = 0.15 h^−1^). Under the latter condition, the cells, furthermore, reached a lower biomass concentration.

The opposite effect of the dissolved oxygen level was found regarding formation of the peptide. The set-up, operated at 30%, produced the least amount (320 BU mL^−1^), even ten-fold less than observed in shake flasks (Fig. 1A). In contrast, the highest amount of pediocin PA-1 (5,590 BU mL^−1^) was formed at the lowest dissolved oxygen level (2.5%). The results identified the control of the DO at 2.5% as optimal for pediocin PA-1 production. Because, growth was already strongly inhibited under these conditions, an even further reduction appeared not promising.

### Medium optimization provides elevated levels of pediocin PA-1 at reduced amount of complex ingredients

So far, pediocin PA-1 production in *C. glutamicum* was based on rich TY medium that contained high levels of expensive ingredients such as yeast extract and tryptone plus glucose. The composition was based on complex media, used before to produce bacteriocins in lactic acid bacteria, well-known hosts to provide this type of product [31]. Towards a simplified and cheaper recipe, we upgraded a commonly used minimal medium for *C. glutamicum* that contained glucose (10 g L^−1^) as carbon source plus mineral salts and vitamins [32] for pediocin PA-1 production. Because it had been reported that the synthesis of bacteriocins benefits from supplementation with yeast extract, whereas tryptone supplements have weaker effects [33], we decided to add 10 g L^−1^ of yeast extract to the minimal formulation. The obtained GY medium exhibited a total carbon content of 0.7%, as inferred from inspection of the individual components [34], 60% less than the original TY medium (total carbon content 1.7%) [23, 34]. When growing strain CR099 *pXMJ19 P*_*tac*_* pedACD*^*Cg*^ in the new GY medium, pediocin PA-1 production was enhanced to 11,056 BU mL^−1^ (Fig. 1C), almost 2.5-fold more than in the original medium. In addition, the novel medium enabled significantly faster growth. The GY medium was therefore kept for further optimization.

### Pediocin PA-1 production in* C. glutamicum* is boosted by acidic pH value

Previously, the pH value was shown to influence the adsorption of bacteriocins to producing cells and, also, the post-translational maturation processes for bacteriocin activation [35]. As example, bacteriocin production was found to be triggered by a pH decrease, and this phenomenon has been also observed for the native pediocin PA-1 producer *P. acidilactici* [36] as well as for other bacteriocins and their native producers [20]. Of note, we recently were able to establish recombinant production of garvicin Q, another class II bacteriocin, with *C. glutamicum*. Considerable levels of garvicin Q were only achieved in minimal medium without urea allowing acidification during cultivation [37]. The pediocin PA-1 production in *C. glutamicum*, conducted in shake flasks on TY medium, revealed the same trend (Additional file 1: Fig. S2A). The initially neutral pH decreased to values below pH 6 during the first 7 h, where it stabilized for the rest of the process. Interestingly, pediocin PA-1 only accumulated in the medium when the pH had become slightly acidic. The same picture resulted for the production in GY medium (Additional file 1: Fig. S2B). The pH decrease was associated to the formation of lactic acid that accumulated in the medium (Fig. 1). Furthermore, online monitoring of the DO indicated that the cells were oxygen-limited during long phases of the process (Additional file 1: Fig. S2A), which apparently triggered formation of the acid [38, 39].

**Fig. 3:**
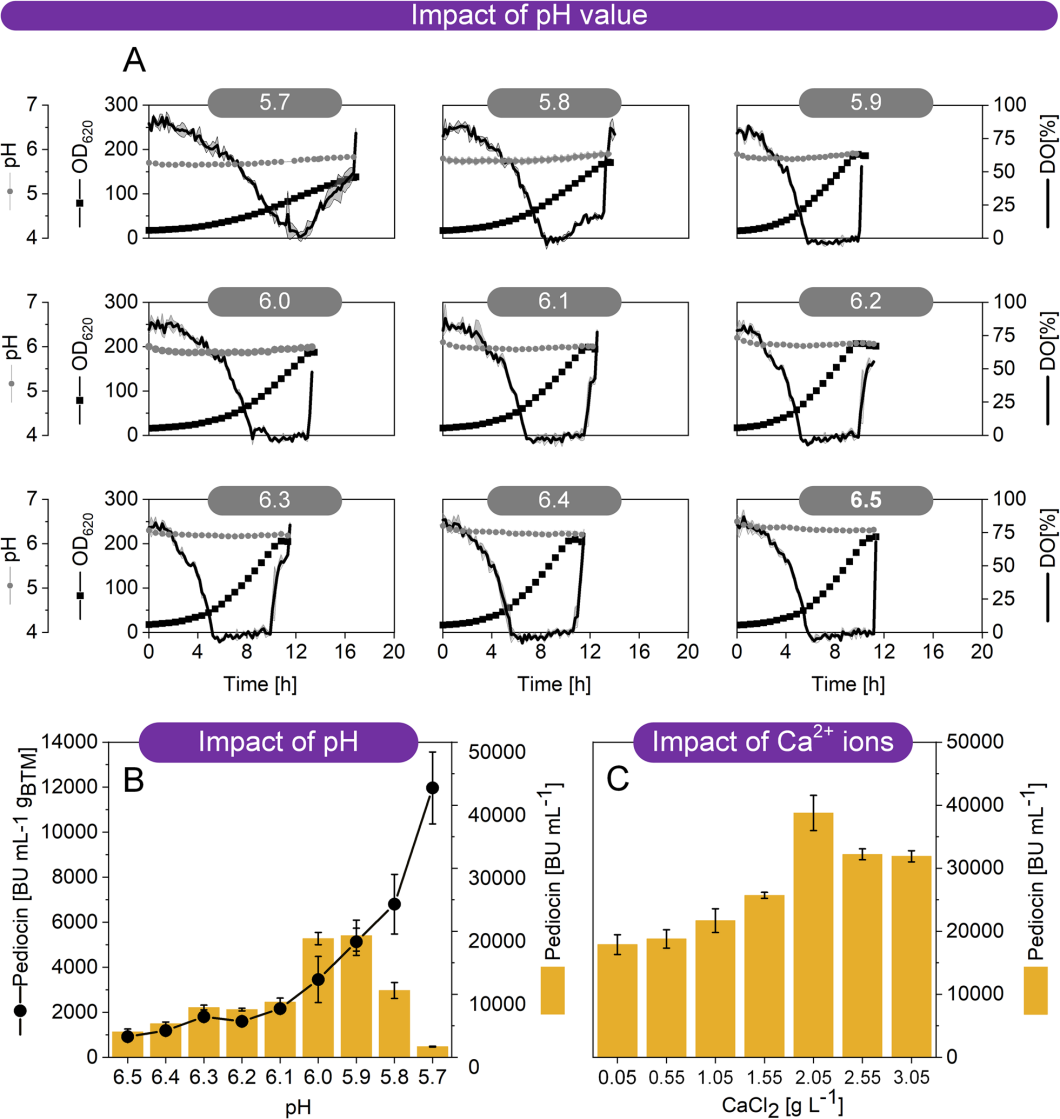
Improvement of pediocin production in recombinant *C. glutamicum* CR099* pXMJ19 P*_*tac*_* pedACD*^*Cg*^ by medium optimization. The cultures were conducted in a miniaturized microtiter plate system with online sensing of cell concentration, pH value, and DO level (**A**). In a first series of experiments, the pH was adjusted to different values between 5.7 and 6.5 using 200 mM MES (**A**). Impact of the pH value on pediocin production (**B**). The data display the final pediocin activity in culture supernatant. In addition, the specific production efficiency is given, normalized to the period of oxygen limitation (required for pediocin accumulation) and the average cell concentration during this phase. In a second series of experiments, the impact of CaCl_2_ on pediocin production was assessed (**C**). Different levels of were supplemented to the medium. The pH was kept at 5.9. The data display the final pediocin activity in culture supernatant. n = 3

To explore the potential of acidic pH for improved pediocin PA-1 formation, the cell factory CR099 *pXMJ19 P*_*tac*_* pedACD*^*Cg*^ was grown on GY medium, buffered with MES at different pH values between 5.7 and 6.5 (Fig. 3A, B). Pre-tests had shown that growth was not possible at more acidic conditions. The cultures were conducted in a microbioreactor using 48-well plates with integrated OD_620_, pH, and DO sensors that allowed precise online monitoring of these parameters. As shown, the pH could be well controlled at each desired set point. Remarkably, the pH value had a strong effect on the final pediocin PA-1 titer, and acidic conditions were found optimal. The specific pediocin PA-1 production, normalized to the phase of oxygen limitation and the corresponding average amount of biomass, was dramatically increased with decreasing pH value. It was highest at pH 5.7, the lowest value that the cells still tolerated. Production was also decreased at more neutral pH values, although the cells grew better under these conditions. On the other hand, cell growth was impaired at two most acidic conditions, pH 5.8 and 5.7. This caused a lower concentration of producing cells and, *inter* alia, an extended period of oxygen excess, negatively affecting the total amount of product formed. That was why, all in all, the maximum production was obtained at pH 5.9 and pH 6.0. Here, a pediocin PA-1 titer of approximately 20,000 BU mL^−1^ was achieved, almost twice as high as before.

### Calcium chloride supplementation at low pH enhances the accumulation of pediocin PA-1 in the culture supernatant

Ca^2+^ ions were shown to improve the secretion of heterologous proteins by *C. glutamicum* [40, 41]. Moreover, we recently observed a pronounced effect of Ca^2+^ ions on the tolerance of *C. glutamicum* to nisin [42]. The addition of CaCl_2_ (2 g L^−1^) to the culture medium significantly increased the resistance of the microbe against the bacteriocin, and it was concluded that this effect was attributed to the Ca^2+^ ions, that masked negative charges of cell wall constituents and thus prevented binding of nisin. Although, *C. glutamicum* was apparently not sensitive to pediocin PA-1 [23], we hypothesized that reduced (or blocked) binding of pediocin PA-1 to the cells could increase the titer in the supernatant. The original GY medium contained 55 mg L^−1^ CaCl_2_. This low level, typically used in defined media to support growth of *C. glutamicum* [43–45], was almost 40-fold lower than the concentration, found effective to affect resistance of the microbe to nisin [42]. Therefore, a next series of cultures was conducted at microbioreactor scale in GY medium, this time buffered at pH 5.9 and containing different levels of CaCl_2_ (0.05–3.05 g L^−1^) (Fig. 3C). Beneficially, pediocin PA-1 production was enhanced up to two-fold by increased CaCl_2_ levels. Maximum pediocin PA-1 activity in the culture supernatant (40,000 BU mL^−1^) was observed at 2 g L^−1^ of CaCl_2_ and above. At this point, we regarded the slightly acidic (pH 5.9) medium, containing glucose, yeast extract, 2 g L^−1^ CaCl_2_ plus other mineral salts and selected vitamins suitable for pediocin PA-1 production in *C. glutamicum*.

### The overexpression of different stress-protecting genes does not further promote growth of *C. glutamicum* at low pH and low oxygen

**Fig. 4:**
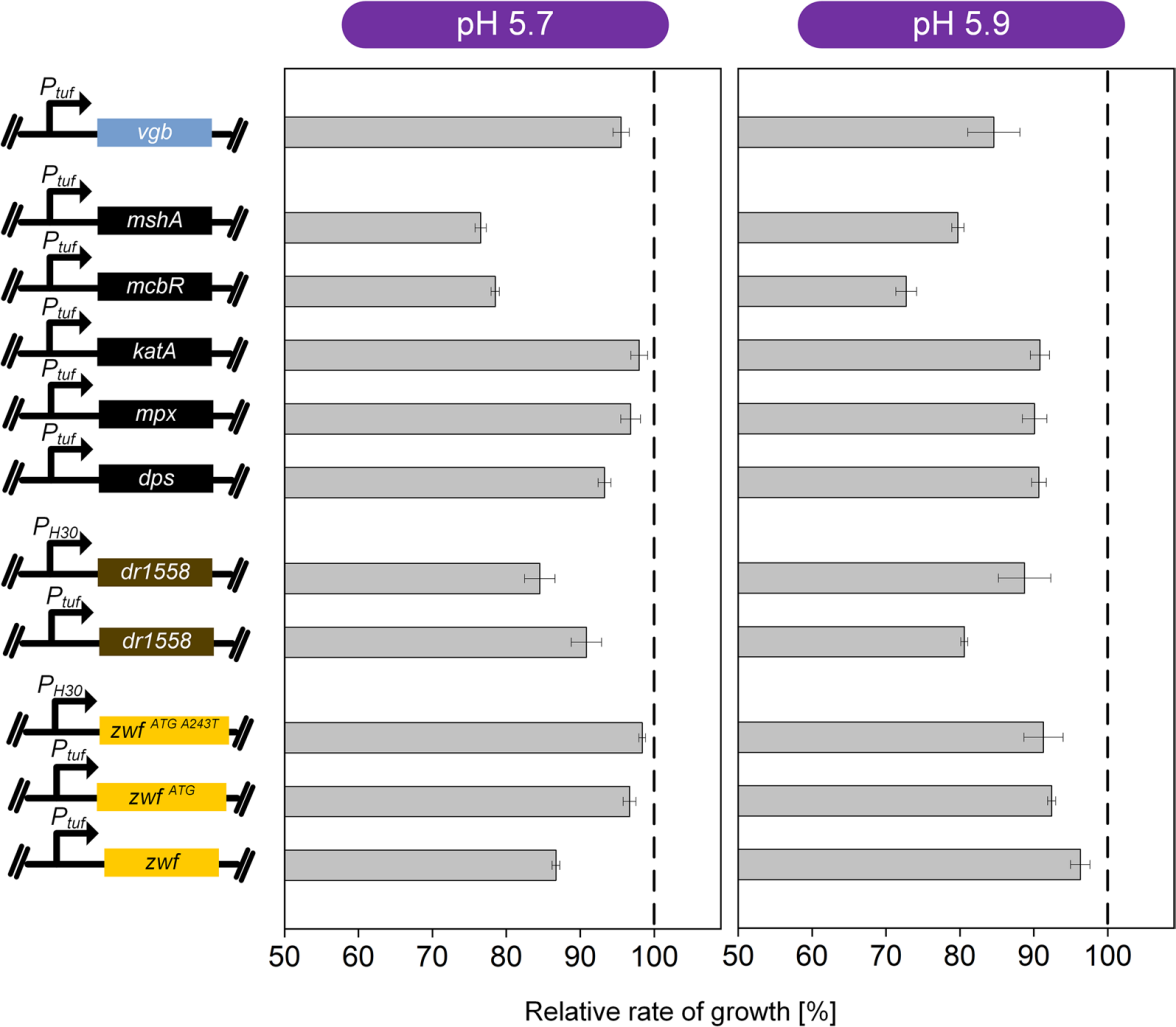
Evaluation of ten gene candidates to support growth of the chassis strain *C. glutamicum* CR099 at pH 5.9 and low oxygen supply. The cultures were conducted in a miniaturized microtiter plate system with online sensing of cell concentration. The different gene candidates were previously studied to increase the tolerance of *C. glutamicum* and encoded for homologous and heterologous proteins: *dps*, iron-storage protein Dps; *mpx*, mycothiol peroxidase; *katA*, catalase; *mcbR*, transcriptional repressor McbR; *mshA*, D-inositol 3-phosphate glycosyltransferase [47–49]; *DR1558*, transcriptional response regulator [50] (Table 2). Each gene was episomally expressed under control of the constitutive promoters *P*_*tuf*_* and P*_*H30*_. A strain expressing the empty vector served as control. n = 3

Although the pediocin PA-1-producing cell factory could grow well at acidic pH and low oxygen level, the optimum production conditions clearly differed from the growth optimum of the microbe which naturally prefers neutral pH and sufficient oxygen supply [46]. We were interested, if growth under pediocin PA-1-producing conditions could eventually be stimulated by the expression of genes, previously found helpful to grow other *C. glutamicum* strains under stress conditions. With regard to low pH, the selected candidates included the *C. glutamicum* genes *dps*, *mpx*, *katA*, *mcbR*, and *mshA* [47–49], as well as the heterologous gene *DR1558* from *D. radiodurans* [50], encoding for homologous and heterologous proteins that had been found beneficial for growth under acidic conditions (Fig. 4). With regard to low oxygen, we chose the gene *vgb* from *Vitreoscilla spp.*, encoding a bacterial hemoglobin, shown to increase oxygen uptake in *C. glutamicum* [51, 52]. Furthermore, we aimed to enhance the supply of the reducing equivalent NADPH, well-known for its importance in the defense of *C. glutamicum* to stress [53]. The latter was realized by over expression of *zwf*, encoding glucose 6-phosphate dehydrogenase at the entry into the PP pathway, *inter alia*, displaying the major route of NADPH supply in *C. glutamicum* [54]. In addition to the native *zwf* gene, we tested two variants for enhanced translation efficiency and enhanced NADPH supply [55, 56]. Each of the ten candidates was individually overexpressed in the chassis strain *C. glutamicum* CR099, using a strain that expressed the empty vector as a control.

All eleven strains were grown in the newly developed production medium at pH 5.7 and pH 5.9 with online monitoring of cell growth. None of the expressed genes, however, could improve the specific growth rate or the biomass formed (Fig. 4). The expression of several genes in fact even reduced the growth ability. For example, overexpression of the native genes *zwf*, *mshA,* and *mcbR* reduced growth. Likewise, DR1558 expression negatively affected cell vitality. Taken together, the unmodified CR099 chassis seemed to work best and was therefore kept.

### Benchmarking the recombinant cell factory under optimum process conditions enables high-efficiency pediocin PA-1 production

**Fig. 5:**
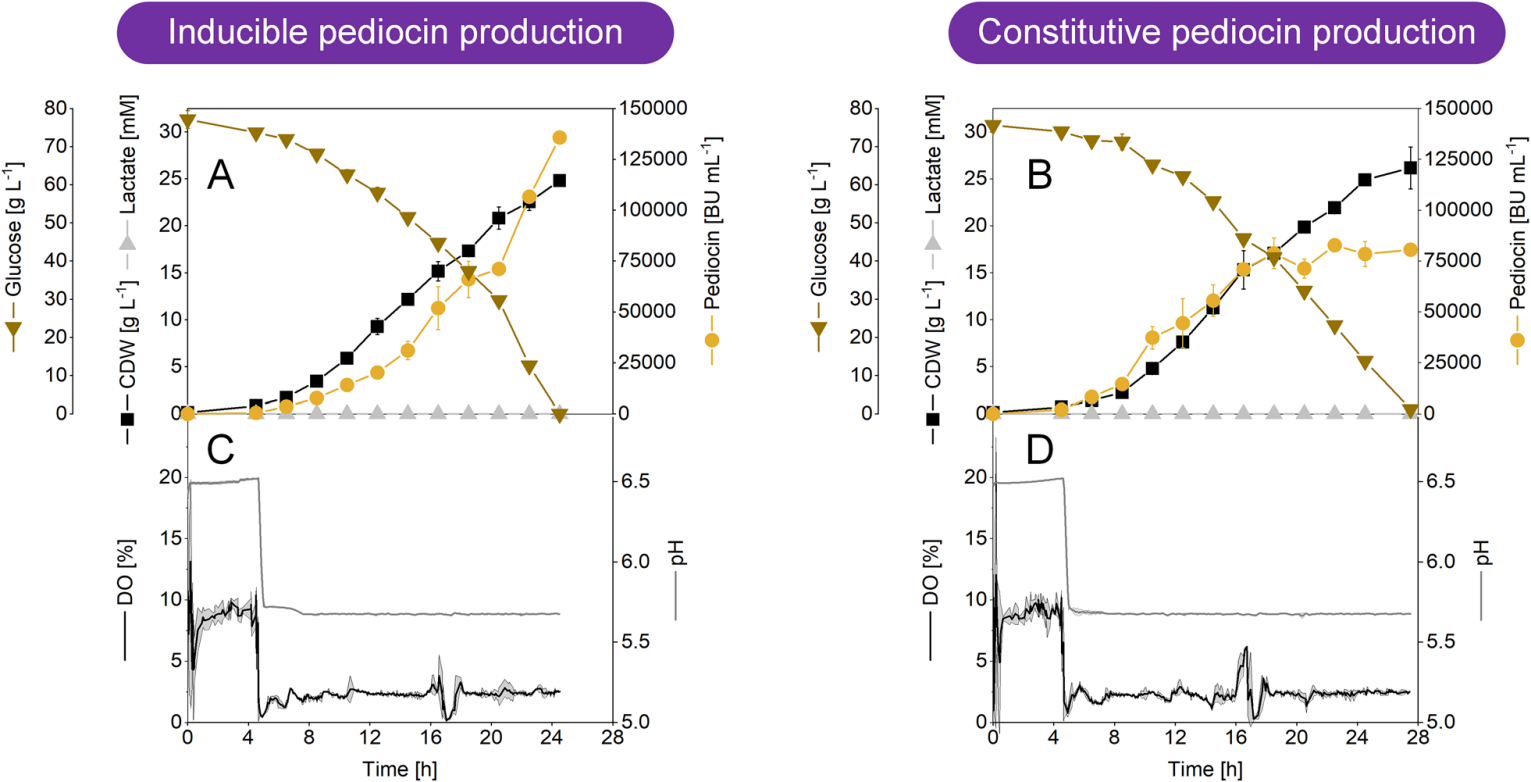
Optimized pediocin PA-1 production in lab scale bioreactors in batch mode using recombinant *C. glutamicum*. As carbon source, the medium contained 80 g L^−1^ of glucose and 15 g L^−1^ of yeast extract. After an initial growth phase (10% DO, pH 6.5), the process was shifted to pediocin PA-1 production (2.5% DO, pH 5.7). The data comprise *C. glutamicum* CR099 *pXMJ19 P*_*tac*_* pedACD*^*Cg*^ which expressed the cluster under control of the *P*_*tac*_ promotor and was induced by 0.2 mM IPTG at process start (**A**, **B**). In addition, *C. glutamicum* CR099 *pClik 5α* *P*_*tuf*_* pedACD*^*Cg*^ was used, expressing the cluster under control of the constitutive *P*_*tuf*_ promotor, so that no inducer was required (C, D)*.* n = 2

Finally, all individual improvements were combined in an optimized process setting. The developed process strategy was benchmarked in lab-scale bioreactors, operated in batch mode with the inducible pediocin PA-1 producer *C. glutamicum* CR099 *pXMJ19 P*_*tac*_* pedACD*^*Cg*^ (Fig. 5A, B). To maximize production, a few changes were made in comparison to the  shake flask set-up. First, the initial glucose concentration was set to 80 g L^−1^ to enable growth to higher cell concentrations, given the fact the *C. glutamicum* copes well with high sugar levels [57]. In addition, the concentration of yeast extract was slightly increased (15 g L^−1^). Second, the medium was buffered, after initial test fermentations had shown that the use of an unbuffered medium resulted in larger fluctuations of the pH (data not shown). Third, the pH value was set to 6.5, while the DO level was controlled at 10% to support cell growth during the initial process phase. Both parameters were supposed to be shifted at a later stage. Fourth, the inducer IPTG was present in the initial medium, after it had turned out that the induced cluster did not affect strain vitality (Table 1).

As intended, the producer CR099 *pXMJ19 P*_*tac*_* pedACD*^*Cg*^ started to grow from early on (Fig. 5A). Within 5 h, the biomass concentration increased to 0.7 g L^−1^, about four-fold. As expected for pH 6.5, pediocin PA-1 formation was negligible during this initial phase. After 5 h, the process conditions were shifted to pH 5.7 and 2.5% DO, because the cells had shown the highest specific production performance under these conditions. It turned out that this set-up allowed highly efficient pediocin PA-1 production. Within only 24 h, the activity in the culture supernatant increased to 135700 BU mL^−1^ (66 mg L^−1^). Towards the end of the process, the cells continued to grow and reached a final biomass concentration of 25 g L^−1^, while glucose was completely consumed. Even though oxygen was limiting conditions, the cells did not form any lactate. This was a remarkable finding given the fact that lactate is the predominant by-product of *C. glutamicum* during growth with glucose under oxygen limiting conditions [58]. Notably, the mutant accumulated high amounts of lactate under the same limiting oxygen level, however at an elevated pH of 6.4 (Fig. 2C). A pH dependence of lactate formation was also obvious from the shake flask cultures, where cells stopped to excrete the acid at a pH below 6.0 (Fig. 1). We conclude that the low pH value of the production process (pH 5.7) abolished lactate formation. From a process point of view, the absence of lactate, a short chain organic acid that is well known for its toxicity to microbes, appeared highly beneficial.

Given the fact, that the immediate induction of the *pedACD*^*Cg*^ cluster at process start apparently had no negative effects, we created the new strain CR099 *pClik 5α P*_*tuf*_* pedACD*^*Cg*^, an alternative producer that expressed the genes constitutively. For expression, we selected the promotor *P*_*tuf*_, the promotor of the gene of elongation factor TU, known to efficiently support gene expression in the microbe [59–61]. When grown under the same conditions, the constitutive producer performed well (Fig. 5C, D). Cells started to grow immediately and handled the shift to acidic pH and low oxygen availability very well. Again, no lactate was formed throughout the whole process. During the first 18 h, pediocin PA-1 production was even stronger than that for the inducible strain. However, during the final stage of the process, the formation of pediocin PA-1 slowed down, despite the cells were still growing and consumed glucose. Finally, an activity of 82,800 BU mL^−1^ (42 mg L^−1^) was achieved.

## Discussion

### Under optimized conditions, ***C. glutamicum*** provides 66 mg L^−1^ of active pediocin PA-1 surpassing previous efforts to derive the bacteriocin almost seven-fold

**Fig. 6:**
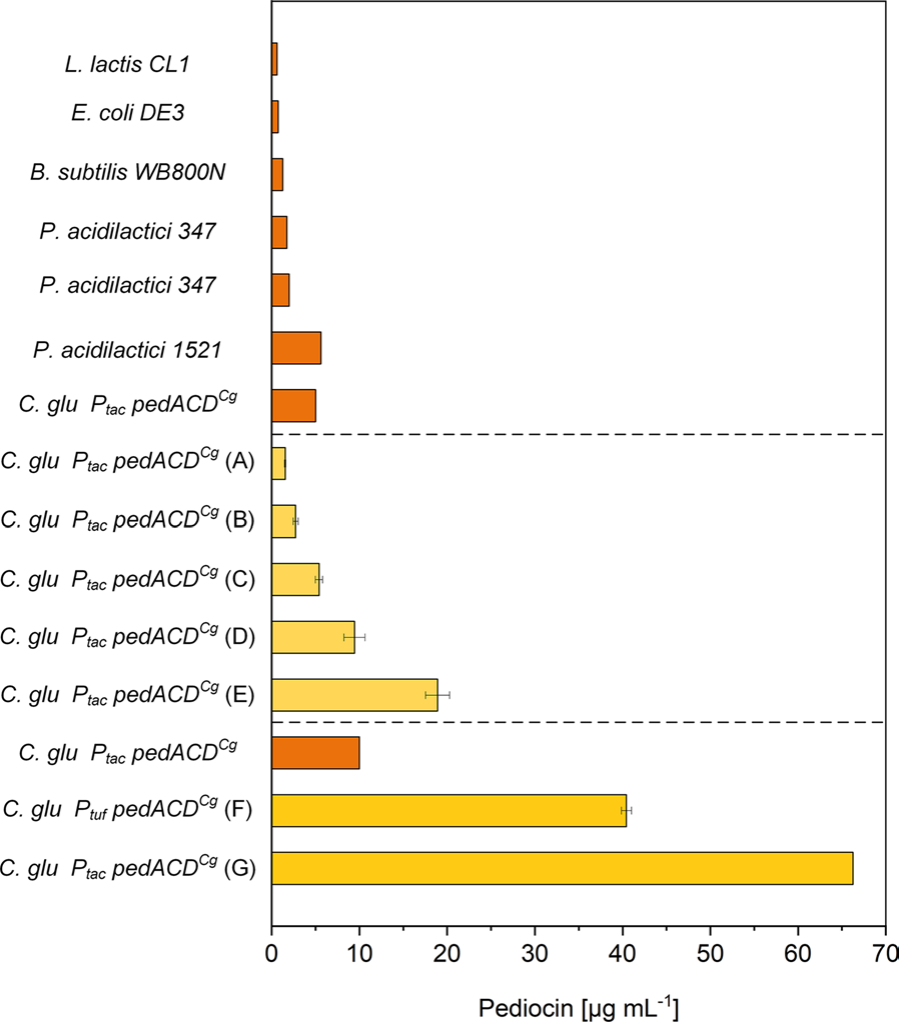
Benchmarking of microbial pediocin PA-1 production in natural and recombinant cell factories. The data show previous efforts (from top to bottom in the upper part) using *L. lactis* CL1 [107], *B. subtilis* [108], *P. acidilactici* 347 [109], *E. coli* producing the M31L version of the peptide [110], *P. acidilactici* 347 [111], *C. glutamicum* CR099 *pXMJ19 P*_*tac*_* pedACD*^*Cg*^ [23], and *P.* *acidilactici* 1521 [112] in shake flasks and test tubes. In addition, the results of the optimization from this work are shown, using *C. glutamicum* CR099 *pXMJ19 P*_*tac*_* pedACD*^*Cg*^. The data comprise (from top to bottom in the middle part) production in in shake flasks using TY medium (**A**), TY medium, at a reduced DO level of 2.5% (**B**), GY medium at reduced nutrient content (**C**), GY medium at pH 5.9 (**D**), and GY medium at pH 5.9 and 2 g L^−1^ CaCl_2_ (**E**). Furthermore, the performance of pediocin PA-1 production in bioreactors is shown. This includes a fed-batch process in TY medium using *C. glutamicum* CR099 *pXMJ19 P*_*tac*_* pedACD*^*Cg*^ [23] and two batch processes in GY medium with 80 g L^−1^ of glucose and 2 g L^−1^ CaCl_2_ from this work, that comprised an initial phase of growth (pH 6.5, 10% DO) and a switch to production (pH 5.7, 2.5% DO) after 4 h using *C. glutamicum* CR099 *pClik P*_*tuf*_* pedACD*^*Cg*^ CR099 with constitutive cluster expression (**F**) and the inducible producer *C. glutamicum* CR099 *pXMJ19 P*_*tac*_* pedACD*^*Cg*^ (**G**). The medium of the letter additionally contained 2 mM IPTG. The pediocin PA-1 concentration obtained with *B. subtilis* was estimated from the reported biological activity [108] using the recently obtained conversion factor [23]

Bacteriocins are a group of antimicrobial peptides that exhibit remarkable activities against dangerous pathogens, including *Staphylococcus*, *Bacillus*, *Listeria,* and *Enterococcus* [62–64]. Pediocin PA-1 is a prominent, commercially relevant bacteriocin [65]. It efficiently combats *L. monocytogenes*, a food-borne pathogen that causes invasive Listeriosis, a severe infection of the bloodstream or the brain in older adults and people with weakened immune systems, leading to death in one out of five cases [7, 66–68]. When listeriosis occurs during pregnancy, it can cause abortion, fetal death, and neonatal morbidity [69]. Although research results regarding the efficiency of pediocins, among other bacteriocins, as bio-preservatives are remarkable and promising, there is substantial reluctance by the industry to commit financially in developing commercial preparations, due to the costly production (low production rates, unstable products and expensive downstream processing) [65]. Here, we demonstrate high-efficiency production of pediocin PA-1 (Fig. 5). As shown, using special producing conditions of low pH, low oxygen level, and elevated amounts of calcium ions, recombinant strains of *C. glutamicum* accumulated pediocin PA-1 up to 66 mg L^−1^, surpassing previous efforts in various producers almost seven-fold (Fig. 6). Hereby, the simplification of the production medium from a costly complex nutrient mixture to a glucose-based formulation with only minor shares of yeast extract enabled production at significantly lower costs (Fig. 1). Beneficially, the creation of a cell factory that expressed the biosynthetic *pedACD* cluster under control of the constitutive *P*_*tuf*_ promotor made it possible to dispense with the addition of IPTG to the production process (Fig. 5), previously needed [23]. IPTG is an efficient molecular inducer for regulating transcriptional activity but suffers from limitations due to toxicity, cost, and culture monitoring [70] so that the demonstrated IPTG-free production of pediocin PA-1 appears attractive. Future fine-tuning of the promotor strength might help to further optimize performance when using constitutive promotors [71]. Taken together, the developed process (Fig. 6) provides a valuable improvement to overcome the present challenges linked to commercial pediocin production [65].

### A special combination of environmental conditions drives the production of active pediocin PA-1 in *C. glutamicum*

Different to most of the products that are made using *C. glutamicum* under aerobic conditions and neutral pH [56, 72, 73], the production of pediocin PA-1 under these conditions was very weak (Fig. 1). In fact, it required the use of a special set-up to drive production of the peptide to high efficiency (Figs. 2, 3, 5). Due to its oxygen sensitivity, active pediocin could not be produced at high aeration, commonly used for the microbe, but required limiting conditions [23] with optimum performance at 2.5% DO (Figs. 2, 5). These microaerobic conditions promoted cell growth better than the simplified control via the shaking rate during flask-based and microbioreactor-based production that always ended up in DO levels of 0% (Figs. 1, 3). A bit surprising, the expression of a mutated M31L-pediocin PA-1 under high oxygen supply provided the peptide only at low level (Additional file 1: Fig. S3), although the variant had been shown to be stable, when stored under these conditions [29]. Eventually, the expression machinery of the host did not well provide the mutant peptide in its active form, or the process environment negatively affected the peptide structure, but more work is needed to clarify this outcome.

An important outcome was the observation that acidic pH and elevated levels of bivalent calcium ions boosted pediocin PA-1 production (Fig. 3). At neutral pH, the cationic peptide [74] interacted with negatively charged residues on the cell wall of *C. glutamicum* [75]. It is well understandable that this interaction caused massive absorption of the peptide to the cells, resulting in apparently low levels of free pediocin PA-1 in the culture supernatant. Indeed, we recently observed that, when incubated *C. glutamicum* ATCC13032 at neutral pH, more than 90% of garvicin Q, another class II bacteriocin, was adsorbed to biomass [37]. A low pH reduced the negative charge of the cell wall by the elevated proton concentrations. In the same direction, the bivalent calcium ions paired with the negative charges on the cell surface. Thus, both low pH and bivalent cations may result in reduced interaction between the cell envelope and cationic peptides. Although definite proof for the exact mechanistic effect of low pH and Ca^2+^ ions on peptide adsorption is still missing, these conditions were highly beneficial for pediocin production. As shown, the specific pediocin production rate, normalized to the biomass, increased almost exponentially with reduced pH and was highest at pH 5.7, the lowest value tested (Fig. 3B). Notably, most small bacteriocins are cationic [76] and therefore similarly absorb, so that acidic conditions appear generally crucial when producing such molecules in *C. glutamicum*. It should, however, be noticed that the acidic environment posed a challenge to the microbe. Growth of *C. glutamicum* was negatively affected already at pH 6, as shown here (Fig. 3) and before [77], while maintenance of the cell structure obviously requires an extracellular pH value above 5.5 [78]. Fortunately, the precise pH control in the bioreactor process and the implementation of an initial phase at pH 6.5 to support growth, allowed to successfully operate at pH 5.7 during later production, close to the growth minimum of the bacterium. Beneficially these conditions fully abolished the formation of lactate, an otherwise undesired by-product.

### *C. glutamicum* shows favorable robustness under the special conditions required for recombinant PA-1 production

The recombinant host was found almost unaffected by the overexpression of the pediocin PA-1 cluster (Fig. 1A,B, Table 1) and, appeared highly vital even in late stages of the production process, where it faced high levels of the antimicrobial peptide, low oxygen availability, and an inhibitory acidic environment (Fig. 5). Notably, *C. glutamicum* has been shown to be robust for the production of a range of demanding chemicals at high concentration, sometimes using quite toxic feedstocks [32, 59, 61, 79, 80]. The microbe performed well under suboptimal growth conditions such as oxygen limitation, required to synthetize lactate [81] and succinate [82], and acidic pH, promoting the production of diaminopentane [50]. In addition, our work demonstrates substantial robustness of *C. glutamicum* to challenging conditions, and that this high naturally existing tolerance makes the bacterium, also, a superior producer of bacteriocins.

It appeared a bit surprising, however, that none of the tested stress-protecting gene candidates promoted growth of the chassis strain CR099 in the acidic oxygen limiting environment but partially even negatively affected growth. An interesting observation was the reduced growth upon stronger expression of the glucose 6-phosphate dehydrogenase gene (Fig. 4, Table 2). As shown before for *C. glutamicum*, this design pushes extra carbon into the pentose phosphate (PP) pathway while reducing the flux through the Emden-Meyerhof-Parnas (EMP) pathway [83]. Efficient growth of *C. glutamicum* under oxygen limitation, as applied here, however, relies on a high EMP pathway flux [81, 84]. It therefore seems that the negative growth effect might be due to an unfavorable pathway flux for the specific conditions used here. In contrast, a higher PP pathway flux was found beneficial under oxidative stress conditions (in the presence of sufficient oxygen), obviously posing a different demand on the cell [53].

**Table. 2:** Impact of recombinant pediocin production in *C. glutamicum* on global gene expression

Locus tag	Gene	Gene description	log2-fold change
Up regulated			
	*pedA*	Pediocin biosynthesis	12.8
	*pedD*	Pediocin biosynthesis	12.8
*CGL_RS05840*		PspC-domain containing protein	3.0
*CGL_RS03930*	*pdxS*	Pyridoxal 5'-phosphate synthase lyase	0.8
*CGL_RS04960*	*aroF*	3-Deoxy-7-phosphoheptulonate synthase	0.7
Down regulated			
*CGL_RS09745*	*bioY*	Biotin transporter	1.6
*CGL_RS09750*		ABC transporter ATP-binding protein	0.6
*CGL_RS10490*		ABC transporter substrate-binding protein	0.6

*C. glutamicum* CR099 *pXMJ19 P*_*tac*_* pedACD*^*Cg*^ was compared with CR099 *pXMJ19* expressing the empty plasmid as control. The cultures were sampled after 13 h (Fig. 1). The data comprise genes that found significantly changed in expression (log_2_-fold change > 1.0). In addition, weakly affected genes are included (1 > log_2_-fold > 0.6). Data significance was verified by a *t* test (p < 0.1). n = 3

In addition, the overexpression of the gene DR1558 from *D. radiodurans* slowed down growth, different to the growth-promoting effect during diaminopentane production in engineered *C. glutamicum* KTCT 1857 [50]. Admittedly, the two set-ups used here, differed in terms of strain background and culture conditions, indicating a complex, yet to be fully explored, impact of the heterologous gene. oge.

## Conclusions

Here, we demonstrated high level production of the antimicrobial peptide pediocin PA-1, a bioactive compound of significant relevance as agent for precision therapy and prevention of infection [4] and, also, for food preservation [5]. The latter covers applications against *Listeria monocytogenes*, a food-borne pathogen of increasing concern [87], explaining the huge interest in pediocin PA-1 production. At present, the compound is not available in pure form, and existing production processes are complex, expensive, and inefficient. After several rounds of optimization, we could show that metabolically engineered *C. glutamicum* accumulated up to 66 mg L^−1^ of active pediocin PA-1, surpassing previous efforts in various microbes to derive the molecule almost seven-fold (Fig. 6). In this regard, our development sets a benchmark towards industrial pediocin PA-1 production.

Traditionally, the microbe is an established workhorse for amino acid manufacturing [88]. Over the years, metabolic engineering efforts have expanded its product portfolio to a range of bulk chemicals [89], materials [90], and fuels [91]. More recent developments, additionally, demonstrated an impressive capability of *C. glutamicum* to synthetize high-value active ingredients for food, feed, human health, and well-being [73], including cell-protective extremolytes [92], flavor compounds [93], pharmaceuticals [94] and nutraceuticals [95]. As shown in this work, *C. glutamicum* appears also capable to take a leading role for the production antimicrobial peptides. The GRAS status, granted to products that are made with the microbe, together with a inherent high metabolic flexibility, genetic accessibility, and process robustness are excellent traits to further drive its development into an antimicrobial peptide production platform and provide many more of these important bioactive compounds.

## Materials and methods

### Microorganisms, plasmids, and genes

*C. glutamicum* ATCC 13032 and the genome-reduced strain *C. glutamicum* *CR099* [96], the recombinant pediocin PA-1 producer CR099 pXMJ19-*P*_*tac*_* pedACD*^*Cg*^*,* and the pediocin PA-1-sensitive assay strains *Listeria innocua LMG2785 pIMK2 (kan*^*R*^*) and LMG2785 pNZ44 (cm*^*R*^*)* were obtained from previous work [23]. Plasmids, based on the episomal vector *pClik5α MCS* [97], were amplified in *E. coli* DH10B (Invitrogen, Carlsbad, CA, USA) [98]. A set of genes was episomally expressed for increased acid tolerance [47–52, 55, 56, 99]. The corresponding plasmids, containing the constitutive promotors *P*_*H30*_ (86 bp) [100] and *P*_*tuf*_ (200 bp) [101], the heterologous genes DR1558 from *D. radiodurans* [50] and *vgb* from *Vitreoscilla* spp. [52], the *C. glutamicum* genes *mcbR*, *katA, mshA, zwf*, the translational start codon variant *zwf *^*ATG*^ [55, 56], and the additionally feedback resistant variant *zwf *^*ATG, A243T*^ [83], respectively, were synthesized from digital sequence information (GenScript, Piscataway, NJ, USA). All strains and plasmids are listed in Table 2.

### Genetic and molecular engineering

The software SnapGene 5.3.2 was used to design strategies for genetic engineering (GSL Biotech, Chicago, IL, USA). For episomal gene expression in *C. glutamicum*, the amplification and assembly of DNA fragments and the amplification, purification, and transformation of the obtained plasmids into *E. coli* and *C. glutamicum* was performed as described previously [57, 71, 102]. Two novel pediocin PA-1 producers were constructed. *C. glutamicum pClik P*_*tuf*_* pedACD*^*Cg*^ was derived by replacing the inducible promotor *P*_*tac*_ by the constitutive *P*_*tuf*_ promotor [61]. *C. glutamicum* pXMJ19 *P*_*tac*_* pedA*^*M31L*^*CD*^*Cg*^ expressed a mutated *pedA*^*M31L*^ gene that exhibited a substitution of l-methionine by l-leucine at position 31 [29]. The desired derivative was created via PCR using primers that contained the point mutation [60]. The primers used are given in the supplementary information (Additional file 1: Table S1).

### Growth media

Different media were used in this work. Pre-cultures were conducted in BHI medium (37 g L^−1^, Brain Heart Infusion, Becton Dickinson, Heidelberg, Germany). For plate cultures, the medium was supplemented with 12 g L^−1^ of agar (Becton Dickinson). For initial pediocin PA-1 production, complex TY medium was used, as described before [23]. It contained 20 g L^−1^ of yeast extract (Becton Dickinson), 15 g L^−1^ of Bacto- tryptone (Becton Dickinson), and 5 g L^−1^ of NaCl. The TY medium was supplemented with 20 g L^−1^ of glucose after 2 h of cultivation [23]. Towards optimized production, a lean medium (designated as GY medium) was developed from a minimal medium recipe [32]. At the start it contained per liter: 10 g of glucose, 15 g of (NH_4_)_2_SO_4_, 10 g of yeast extract (Becton Dickinson), 1 g of NaCl, 0.2 g of MgSO_4_ 7H_2_O, 55 mg of CaCl_2,_ 20 mg of FeSO_4_ 7H_2_O, 0.5 mg of biotin,1 mg of thiamin HCl, 1 mg of calcium panthothenate, 10 mL of a 100 × trace element solution (200 mg L^−1^ FeCl_3_ 6H_2_O, 200 mg L^−1^ MnS04 H_2_O, 50 mg L^−1^ ZnSO_4_ 7H_2_O, 20 mg L^−1^ CuCl_2_ 2H_2_O, 20 mg L^−1^ Na_2_B_4_O_7_ 10H_2_O, 10 mg L^−1^ (NH_4_)_6_Mo_7_O_24_ 4H_2_O, pH 1.0), and 30 µg L^−1^ of 3,4-dihydroxybenzoic acid. During the study, the GY medium was optimized, including the use of 200 mM MES buffer at pH values between 5.7 and 6.5 and supplementation with CaCl_2_ up to 3 g L^−1^. Further details are given below. For plasmid maintenance, the medium was supplemented with kanamycin (50 µg mL^−1^) or chloramphenicol (12.5 µg mL^−1^). When growing inducible production strains, IPTG was added to the culture from a filter-sterilized stock after 2 h to a final level of 0.2 mM.

### Medium screening in a microbioreactor

Medium tests were conducted in a microbioreactor with on-line sensing of growth, dissolved oxygen (DO), and pH-value (BioLector I, 700 rpm, 30 °C, 85% humidity, Beckman Coulter, Baesweiler, Germany) using 48-well flower plates, filled with 1 mL medium per well (MTP-48-BOH1, Beckman Coulter) [80]. The experiments (n = 3) were inoculated from a pre-culture in BHI medium, grown overnight in baffled shake flasks (10% filling volume) at 30 °C and 230 rpm (Infors HT Multitron, Bottmingen, Switzerland) and harvested by centrifugation (3 min, 8800 ×g, room temperature). When growing inducible production strains, IPTG was added to the culture from a filter-sterilized stock after 2 h to a final level of 0.2 mM.

### Process development in lab scale bioreactors

The impact of the dissolved oxygen (DO) level on production was studied in 1 L stirred tank bioreactors, controlled by the DASGIP control software (SR0700ODLS, Eppendorf SE, Hamburg, Germany). Each reactor was filled with 300 mL TY medium. The inoculum was prepared in two steps. First, cells were grown overnight in BHI medium, harvested as described above, and used to inoculate a second pre-culture in TY medium, which was incubated under the same conditions, harvested during the mid-exponential phase (3 min, 8800 ×g, room temperature) and used to inoculate the process to an initial OD_660_ of 0.5. The temperature was set to 30 °C (CWD4 Bioblock, Eppendorf SE). Electrodes were used to monitor the DO (VisiFerm DO 225, Hamilton, Höchst, Germany) and the pH value (405-DPAS-SC-K8S/225, Mettler Toledo, Giessen, Germany). During the process, the pH value was maintained at pH 6.5 by automatic addition of 6 M NaOH and 6 M HCl, respectively. In three different setups, the DO level was controlled at 2.5%, 5%, and 30%, respectively, by adjustment of the stirrer speed and the rate of aeration. Pediocin PA-1 production was induced 2 h after inoculation (0.2 mM IPTG). At the same time point, glucose (20 g L^−1^) was added. Each condition was evaluated as single replicate.

### Pediocin PA-1 production in shake flasks

The inoculum was prepared using two subsequent steps. The first pre-culture, inoculated from a BHI agar plate pre-incubated for two days at 30 °C, was grown overnight in BHI medium in baffled shake flasks (10% filling volume) at 30 °C and 230 rpm (Infors HT Multitron), harvested (3 min, 8800 ×*g*, room temperature) and used to inoculate the second pre-culture which was based on the medium that was later used for the main culture (TY or GY medium) and was grown under the same conditions. Cells were harvested during the mid-exponential phase (3 min, 8800 ×g, room temperature) and used to inoculate the main cultures to an initial OD_660_ of 0.5. The main cultures were incubated in unbaffled shake flasks on an orbital shaker at a low shaking rate (30 °C, 130 rpm, 10% filling volume, Infors HT Multitron) [23]. In selected cases, the cultures were grown in unbaffled flasks with optical sensors for on-line monitoring of pH and DO [103] using the PreSens SFR system (PreSens, Regensburg, Germany). Pediocin PA-1 production was induced 2 h after inoculation (0.2 mM IPTG). When growing cells in TY medium, glucose (20 g L^−1^) was additionally supplemented at this time point. All experiments were conducted in triplicate.

### Pediocin PA-1 production in lab-scale bioreactors

The production of pediocin PA-1 was demonstrated in 1 L bioreactors (SR0700ODLS, Eppendorf), controlled by the DASGIP control software (Eppendorf). Each reactor was filled with 300 mL medium, buffered at pH 6.5 with 200 mM MES. The medium contained per liter: 80 g of glucose, 15 g of (NH_4_)_2_SO_4_, 15 g of yeast extract (Becton Dickinson), 1 g of NaCl, 0.2 g of MgSO_4_ 7H_2_O, 2 g CaCl_2,_ 20 mg of FeSO_4_ 7H_2_O, 0.5 mg of biotin,1 mg of thiamin HCl, 1 mg of calcium panthothenate, 10 mL of a 100 × trace element solution (200 mg L^−1^ FeCl_3_ 6H_2_O, 200 mg L^−1^ MnS04 H_2_O, 50 mg L^−1^ ZnSO_4_ 7H_2_O, 20 mg L^−1^ CuCl_2_ 2H_2_O, 20 mg L^−1^ Na_2_B_4_O_7_ 10H_2_O, 10 mg L^−1^ (NH_4_)_6_Mo_7_O_24_ 4H_2_O, pH 1.0), 30 µg L^−1^ of 3,4-dihydroxybenzoic acid, and 0.2 mM IPTG. The temperature was controlled at 30 °C (CWD4 Bioblock, Eppendorf). Electrodes were used to monitor the DO level (VisiFerm DO 225, Hamilton) and the pH value (405-DPAS-SC-K8S/225, Mettler Toledo). The two parameters were controlled by the automatic addition of 7.3 M NH_4_OH and 6 M NaOH and the adjustment of stirrer speed and aeration rate, respectively. During the first 4 h, the process was operated at pH 6.5 and a DO level of 10%. For the rest of the process, the pH was controlled at 5.7, while the DO level was reduced to 2.5%. The inoculum was prepared via two pre-culture steps, as described above, and used to inoculate the process to an initial OD_660_ of 0.5. The processes were conducted as duplicate.

### Quantification of the cell concentration

The optical density (OD_660_ ) in shake flask and bioreactor cultures was monitored off-line at 660 nm, using a spectrophotometer. A correlation between cell dry weight (CDW) and optical density (CDW [g L^−1^] = 0.32 × OD_660_) was taken from previous work [44] to infer CDW concentrations from OD_660_ readings. In microbioreactor cultures, the photometric analysis was conducted on-line at 620 nm.

### Quantification of glucose

Glucose was quantified by HPLC (1260 Infinity Series, Agilent Technologies, Santa Clara, CA, USA), equipped with a MetaCarb 87 C column (7,8 × 300 mm, Agilent Technologies) as stationary phase that was heated to 80 °C. Deionized water served as mobile phase (1 mL min^−1^) [104]. The refractive index was used for detection (1260 RID G1362A, Agilent Technologies), and external standards were used for quantification.

### Quantification of lactate

Lactate was quantified by HPLC (1260 Infinity Series, Agilent Technologies, Santa Clara, CA, USA), using an ion-moderated partition column (Aminex HPX-87H, 300 × 7.8 mm, 45 °C, Bio-Rad, München, Germany) and isocratic elution with 12 mM H_2_SO_4_ (0.5 mL min^−1^). The refractive index was used for detection (1260 RID G1362A, Agilent Technologies), and external standards were used for quantification.

### Quantification of pediocin PA-1 activity

The biological activity of pediocin PA-1 was determined using a growth inhibition assay [23, 105]. The sensor strains *L. innocua* *pIMK2* and *L. innocua* *pNZ44* were grown overnight in glass tubes (filled 20% with BHI medium) on a rotary shaker (37 °C, 230 rpm, Infors HT Multitron). The samples (culture supernatant) were stepwise diluted with BHI medium in a 96-well microtiter plate. This yielded a twofold dilution series (100 µL for each dilution). In parallel, the *L. innocua* cultures were diluted 1:25-fold in fresh BHI medium, and the obtained suspension was mixed 1:1 with each diluted sample. The filled microtiter plate was incubated for 6 h (37 °C, 230 rpm, Infors HT Multitron). Afterwards, the cell concentration in each well was quantified at 595 nm (Labsystems iEMS Reader MF, Thermo Fisher, Waltham, MA, USA). The pediocin PA-1 activity was then estimated from the growth inhibition data [105], using software-based parameter fitting with the sigmoidal dose response tool (Origin 2021, Northampton, UK). Throughout this study, the activity is given as bacteriocin units per mL (BU mL^−1^). Using the recently determined specific biological activity of purified, commercial pediocin PA-1 [23], allowed to infer peptide concentrations from biological activity measurement.

### Global gene expression profiling

One-color DNA microarrays were used for global gene expression profiling of *C. glutamicum* [106]. In short, custom-made microarrays (8 ×15 K) with 8990 probes were designed with the eArray online tool (Agilent Technologies). The final array covered the entire genome of *C. glutamicum* ATCC 13,032 (entry number: # T00102, KEGG database), the genes *pedA* and *pedD* from the heterologous pediocin PA-1 operon, and 60 Agilent-based quality control probes (SurePrint G3 Custom GE 8 × 60 K, Agilent Technologies). Each gene was represented by three different 45–60 bp probes. Each probe was applied as 6 replicates and randomized to feature locations on the slide. Prior to analysis, cells were quickly harvested (1 min, 13,000 ×g, RT). The obtained pellet was immediately transferred into liquid nitrogen, followed by RNA isolation (RNeasy Mini Kit, Qiagen, Hilden, Germany). Concentration and purity of the isolated total RNA were analyzed by absorption measurement (NanoDrop 1000, PEQLAB Biotechnology GmbH, Erlangen, Germany). Furthermore, the RNA integrity was determined using semi-automatized chip-based electrophoresis (RNA 6000 Nano Kit, 2100 Bioanalyzer System, Agilent Technologies). All samples exhibited an RNA integrity number of > 9.8. Subsequently, 50 ng of total RNA was converted into Cy3-labeled antisense cRNA (Low Input Quick Amp WT Labeling kit, Agilent Technologies). In all cases, the reaction yielded > 825 ng cRNA with a Cy3-activity of > 15 pmol per µg RNA (NanoDrop 1000, PEQLAB Biotechnology GmbH). Then, 600 ng of labeled cRNA was fragmented and hybridized (Gene Expression Hybridization Kit (Agilent Technologies). For hybridization onto the microarray, 40 µL of the prepared cRNA solution was applied in between a gasket slide and the array slide using the SureHyb chamber (G2534A, Agilent Technologies). The paired slides were incubated in a hybridization oven at 65 °C for 17 h (G2545A, Agilent Technologies). Slide holders (G2505-60525, Agilent Technologies) were used to transfer the paired slides into the SureScan microarray scanner cassette (G2600D, Agilent Technologies). Afterwards, the array was scanned (SureScan Microarray Scanner G4900DA, SureScan Microarray Scanner Control Software, Agilent Technologies). Signals were recorded according to the AgilentG3_GX_1 color scanner protocol. Data were extracted by the microarray and feature extraction software (version 12.1.1.1, Agilent Technologies). The software GeneSpring (version 14.9, Agilent Technologies) was used for data visualization. For statistical analysis, a moderated *t* test was applied (Smyth, 2004), considering asymptotic computation of p-values adjusted for multiple testing according to the Benjamini–Hochberg method and a q-value cut-off of 0.05 (Benjamini and Hochberg, 1995). The data were then filtered for genes with a log2-fold change ≥ 1 (p-value ≤ 0.1). RNA extraction and analysis were conducted in biological triplicate for each strain. The entire transcriptome data set is available at GEO (GSE220713).

## Supplementary Information

**Additional file 1 Figure S1.** Principal component analysis of the transcriptome data set comprising the pediocin producer *C. glutamicum CR099 pXMJ19 Ptac pedACD *and its reference *C. glutamicum CR09 pXMJ19*, expressing the empty plasmid. n=3.** Figure S2.** Pediocin production in recombinant *C. glutamicum CR099 pXMJ19 Ptac pedACDCg *using TY medium (**A**). Glucose (20 g L-1) was added after 2 h. Pediocin production was induced, also after 2 h, by the addition of 0.2 mM IPTG. During the process, pH value and DO were monitored online. GY medium based pediocin production with additional pH monitoring (**B**). n=3. **Figure S3.** Pediocin production in recombinant *C. glutamicum *CR099 *pEKEx2 Ptac pedAM31LCDCg *using TY medium in baffled shake flasks (aerobic conditions). Glucose (20 g L-1) was added after 2 h. Pediocin production was induced, also after 2 h, by the addition of 0.2 mM IPTG. The used plasmid is a well-established vector for *C. glutamicum *[1]. n=3. **Table S1.** Primers for strain construction.

## Data Availability

The dataset(s) supporting the conclusions of this article are all included within the article.
