# Supplementary material for: High-efficiency production of the antimicrobial peptide pediocin PA-1 in metabolically engineered Corynebacterium glutamicum using a microaerobic process at acidic pH and elevated levels of bivalent calcium ions
Source: Microb Cell Fact. 2023 Feb 27;22:41. doi: 10.1186/s12934-023-02044-y (PMC9969654; doi:10.1186/s12934-023-02044-y)
Supplement: Supplementary file 1 — Additional file 1 Figure S1. Principal component analysis of the transcriptome data set comprising the pediocin producer C. glutamicum CR099 pXMJ19 Ptac pedACD and its reference C. glutamicum CR09 pXMJ19, expressing the empty plasmid. n=3. Figure S2. Pediocin production in recombinant C. glutamicum CR099 pXMJ19 Ptac pedACDCg using TY medium (A). Glucose (20 g L-1) was added after 2 h. Pediocin production was induced, also after 2 h, by the addition of 0.2 mM IPTG. During the process, pH value and DO were monitored online. GY medium based pediocin production with additional pH monitoring (B). n=3. Figure S3. Pediocin production in recombinant C. glutamicum CR099 pEKEx2 Ptac pedAM31LCDCg using TY medium in baffled shake flasks (aerobic conditions). Glucose (20 g L-1) was added after 2 h. Pediocin production was induced, also after 2 h, by the addition of 0.2 mM IPTG. The used plasmid is a well-established vector for C. glutamicum [1]. n=3. Table S1. Primers for strain construction. [file 12934_2023_2044_MOESM1_ESM.pdf]

Supplementary File to

High-efficiency production of the antimicrobial peptide pediocin PA-1 in metabolically engineered C*orynebacterium glutamicum* using a microaerobic process at acidic pH and elevated levels of bivalent calcium ions

Submitted for publication to Microbial Cell Factories

Jens Christmann[a], Peng Cao[a], Judith Becker[a], Christian K. Desiderato[b], Oliver Goldbeck[b], Christian U. Riedel[b], Michael Kohlstedt[a], and Christoph Wittmann[a*]

[a] Institute for Systems Biotechnology, Saarland University, Saarbrücken, Germany

[b] Institute of Microbiology and Biotechnology, University of Ulm, Ulm, Germany

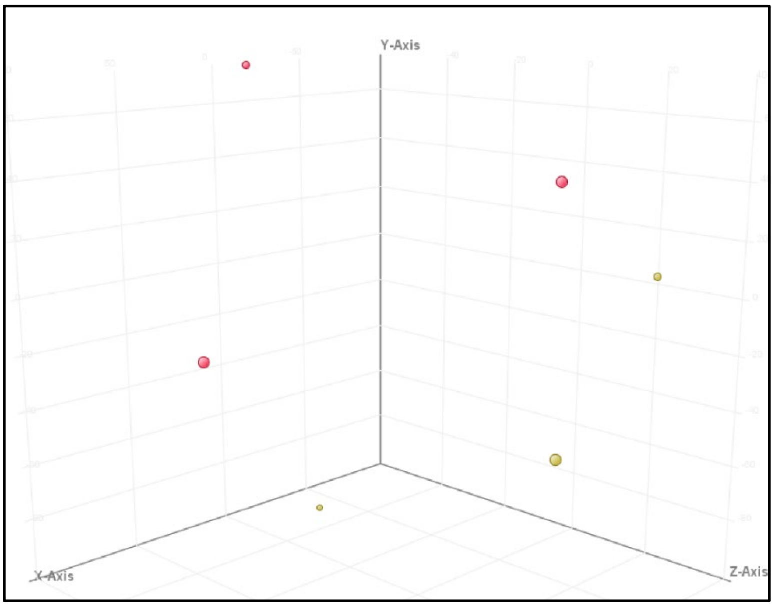

Figure S1*:* Principal component analysis of the transcriptome data set comprising the pediocin producer *C. glutamicum CR099 pXMJ19* P_{tac} *pedACD* and its reference *C. glutamicum CR09 pXMJ19*, expressing the empty plasmid. n=3.

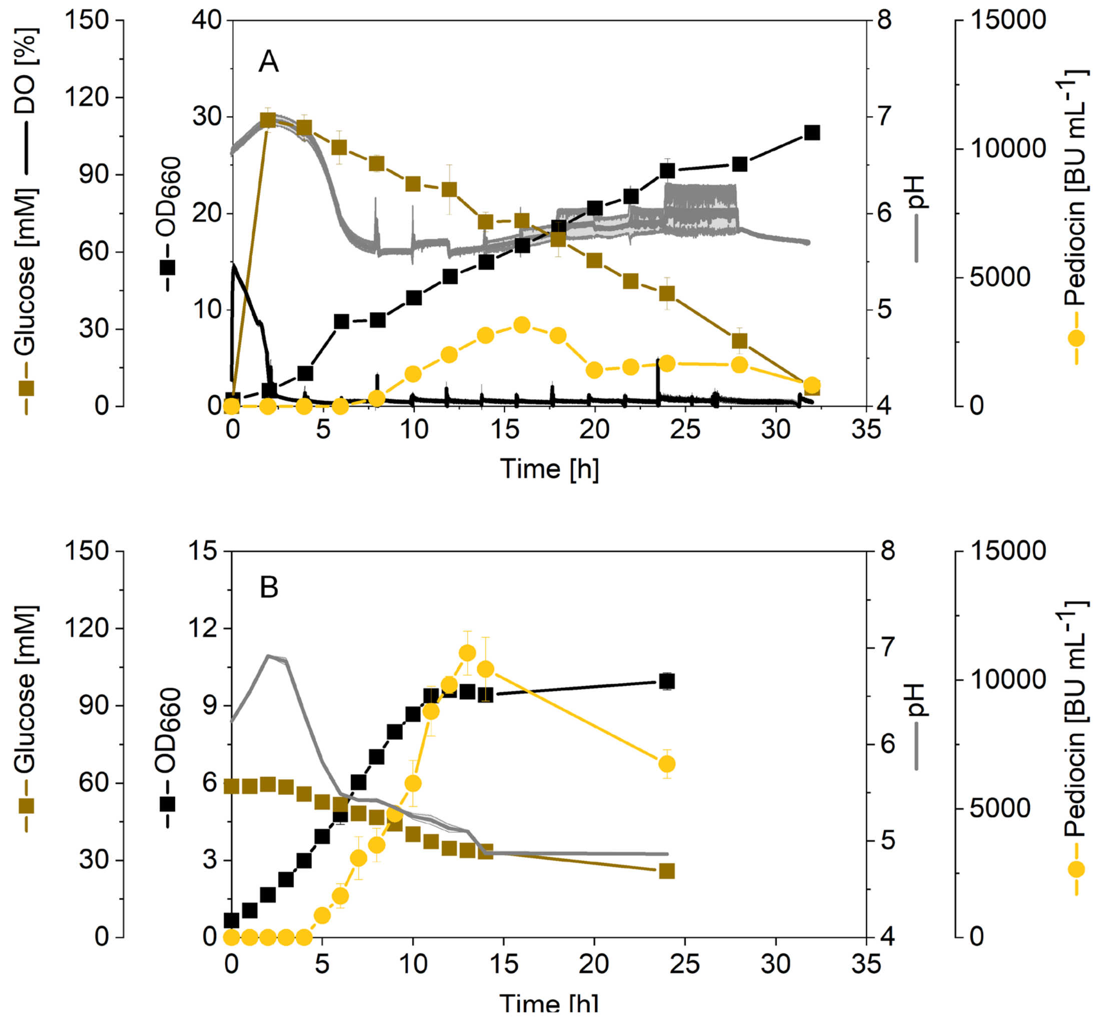

Figure S2: Pediocin production in recombinant *C. glutamicum CR099 pXMJ19* P_{tac} *pedACD*^{Cg} using TY medium (A). Glucose (20 g L^{-1}) was added after 2 h. Pediocin production was induced, also after 2 h, by the addition of 0.2 mM IPTG. During the process, pH value and DO were monitored online. GY medium based pediocin production with additional pH monitoring (B). n=3.

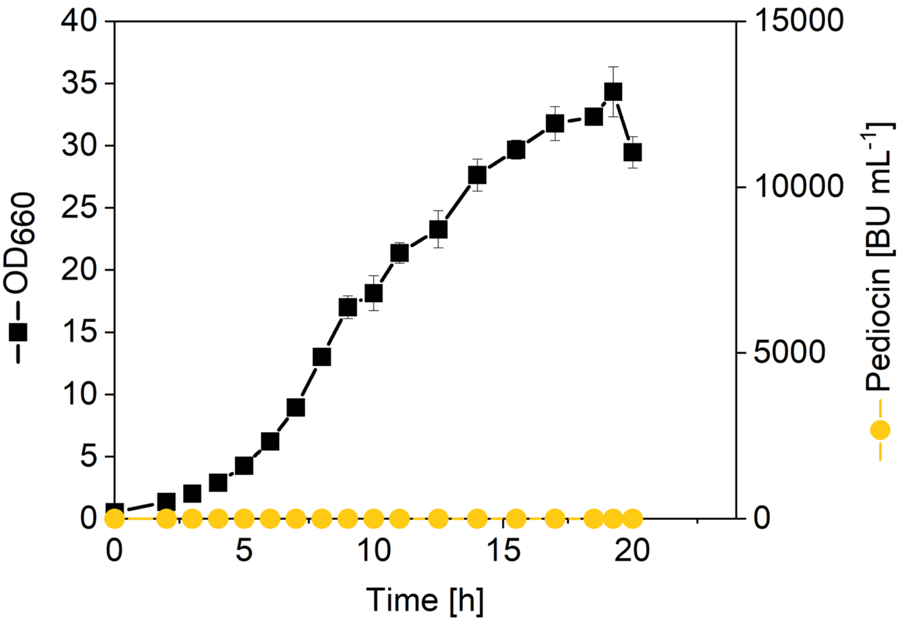

Figure S3: Pediocin production in recombinant *C. glutamicum* CR099 *pEKEx2* P_{tac} *pedAM31LCD*^{Cg} using TY medium in baffled shake flasks (aerobic conditions). Glucose (20 g L^{-1}) was added after 2 h. Pediocin production was induced, also after 2 h, by the addition of 0.2 mM IPTG. The used plasmid is a well-established vector for *C. glutamicum* [1]. n=3.

Table S1: Primers for strain construction.

Primer	Sequence	Annealing T [C°]
Primer 1	GAAGTCCAGGAGGACATACAATGAAGAAGATTGAG AAGCTGACCGA	59
Primer 2	CGCTTCCTTTAGCAGCCCGCTAGCGGATCATTTAAA TCCCTTATTCCTGGTTATGAATGAGGCGTGC	59
Primer 3	GCTCTTCTGCGTTAATTAACAATTGGGATCCTCTAG ACCCTGGCCGTTACCCTGCGAA	59
Primer 4	AGCTTCTCAATCTTCTTCATTGTATGTCCTCCTGGAC TTCGT	59

Literature

1. Bakkes PJ, Ramp P, Bida A, Dohmen-Olma D, Bott M, Freudl R: **Improved pEKEx2-derived expression vectors for tightly controlled production of recombinant proteins in *Corynebacterium glutamicum*.** *Plasmid* 2020, **112:**102540.
